# The TRICLOBS Dynamic Multi-Band Image Data Set for the Development and Evaluation of Image Fusion Methods

**DOI:** 10.1371/journal.pone.0165016

**Published:** 2016-12-30

**Authors:** Alexander Toet, Maarten A. Hogervorst, Alan R. Pinkus

**Affiliations:** 1 TNO, Soesterberg, The Netherlands; 2 Air Force Research Laboratory, Wright-Patterson AFB, Ohio, United States of America; West Virginia University, UNITED STATES

## Abstract

The fusion and enhancement of multiband nighttime imagery for surveillance and navigation has been the subject of extensive research for over two decades. Despite the ongoing efforts in this area there is still only a small number of static multiband test images available for the development and evaluation of new image fusion and enhancement methods. Moreover, dynamic multiband imagery is also currently lacking. To fill this gap we present the TRICLOBS dynamic multi-band image data set containing sixteen registered visual (0.4–0.7μm), near-infrared (NIR, 0.7–1.0μm) and long-wave infrared (LWIR, 8–14μm) motion sequences. They represent different military and civilian surveillance scenarios registered in three different scenes. Scenes include (military and civilian) people that are stationary, walking or running, or carrying various objects. Vehicles, foliage, and buildings or other man-made structures are also included in the scenes. This data set is primarily intended for the development and evaluation of image fusion, enhancement and color mapping algorithms for short-range surveillance applications. The imagery was collected during several field trials with our newly developed TRICLOBS (TRI-band Color Low-light OBServation) all-day all-weather surveillance system. This system registers a scene in the Visual, NIR and LWIR part of the electromagnetic spectrum using three optically aligned sensors (two digital image intensifiers and an uncooled long-wave infrared microbolometer). The three sensor signals are mapped to three individual RGB color channels, digitized, and stored as uncompressed RGB (false) color frames. The TRICLOBS data set enables the development and evaluation of (both static and dynamic) image fusion, enhancement and color mapping algorithms. To allow the development of realistic color remapping procedures, the data set also contains color photographs of each of the three scenes. The color statistics derived from these photographs can be used to define color mappings that give the multi-band imagery a realistic color appearance.

## Introduction

### The significance of image fusion

Night vision cameras are a vital source of information for a wide-range of critical military and law enforcement applications such as surveillance, reconnaissance, intelligence gathering, and security [1, 2]. Currently, most night-time imaging systems are either low-light-level cameras which amplify reflected visible (0.4–0.7μm) to near-infrared (NIR, 0.7–1.0μm) light or thermal, long-wave infrared (LWIR, 8–14μm) cameras which convert thermal energy into a visible image. Because these systems operate in different spectral bands they typically represent different aspects of a scene. For instance, after a period of extensive cooling (e.g., after a long period of rain or early in the morning) the background (i.e., vegetation or soil areas, buildings and other manmade objects) of an outdoor scene may be represented in full detail in the visible bands, but may be much less detailed in the infrared bands due to the low thermal contrast in the scene. On the other hand, objects like vehicles or human beings, which often have an appreciable temperature contrast with their surroundings, will typically be shown with high contrast in the infrared bands. They may be (nearly) invisible (camouflaged-) in the visible bands when their luminance and/or color approaches that of their surroundings. In such cases the thermal component of a fused visible/thermal image may help to detect and localize targets in the context provided by the visible component [2] and contribute to situational awareness [3]. In addition, appropriate color mappings may serve to give multi-band night-vision imagery an intuitive color appearance, which may in turn lead to faster and more accurate scene recognition [4, 5]. Examples of observational tasks that will evidently benefit from realistically rendered fused multi-band imagery are navigation and surveillance tasks.

The increasing availability and deployment of imaging sensors operating in multiple spectral bands [6–16], in combination with lenses that cover a broad spectral range [17] and dedicated image fusion hardware [18–21] has led to a steady stream of publications on methods that combine or fuse the signals from these sensors for viewing by human operators. The goal of these studies is to develop algorithms that effectively combine the different complementary and partially redundant spectral bands to visualize information that is not directly evident from each of the individual input images (i.e., the sum should be more than its parts). Some potential benefits of image fusion are: wider spatial and temporal coverage, decreased uncertainty, improved reliability, and increased system robustness. Image fusion has important applications for situational awareness [3], surveillance [22], target tracking [23], intelligence gathering [24], concealed weapon detection [25–30], detection of abandoned packages [31] and buried explosives [32], and face recognition [33, 34]. In the context of several allied Soldier Modernization Programs (SMPs), image fusion has significantly gained importance [19], particularly for application in head-borne systems [35–37]. Other important image fusion applications are found in industry, art analysis [38], agriculture [39], remote sensing [40–43], and medicine [44–47] (for a survey of different applications of image fusion techniques see [1, 48]).

### The state of the art in multiband nighttime image fusion

The increasing availability of sensors operating at low light levels and in multiple spectral bands has spurred the development of image fusion and enhancement algorithms for surveillance and navigation applications. The general aim is to provide imagery that is both rich in information content (more informative than the individual bands), easy to interpret (ergonomic in a cognitive sense) as well as robust against degradation of environmental conditions and/or sensor performance. To this end many different image fusion techniques have been proposed and new studies appear regularly (for a recent review see [49]). Most methods apply fusion to combine context information from the Visual band with the LWIR band [50–63]. Some fusion methods employ the NIR channel to enhance contrast in the Visual image, exploiting the high permeability of NIR against atmospheric haze [64–66]. Other fusion methods use statistical information [67–69] color lookup tables [70, 71], histogram matching techniques [72] or color transforms [73] to give fused multiband imagery a realistic color appearance by transferring the color characteristics of associated visible color imagery. All methods implicitly assume the availability of spatially registered multiband imagery. Most of the aforementioned fusion methods [50–54, 56–62, 69, 73] were developed with the limited set of static registered multiband (Visual, NIR, LWIR) imagery which we provided earlier [74, 75]. None of the methods discussed have been applied to dynamic multiband image sequences, most likely because of this type of imagery is currently not publicly available.

### The need for registered multi-band imagery

Despite of the ongoing interest in the fusion of multi-band (specifically visual, NIR and LWIR) images for surveillance applications and the steady stream of publications in this area, there is only a very small number of static registered multi-band test images (and a total lack of dynamic image sequences) publicly available for the development and evaluation of image fusion algorithms (e.g., [76]). Moreover, there is no central repository from which these images can be obtained. (The site www.ImageFusion.org, which had provided some multispectral imagery, closed several years ago, although recent publications still refer to this site). To the best of our knowledge there are currently only two dedicated image data sets for the development of image fusion techniques that are available from a public repository. The first is the TNO Image Fusion Data Set [74]. It contains only a limited set of static multispectral (intensified visual, NIR, and LWIR) nighttime imagery of military-relevant scenarios. The second dataset is the Kayak image fusion sequence [75]. It provides registered intensified visual, mid-wave IR (3–5μm) imagery, and LWIR dynamic imagery depicting three kayaks approaching a shore in a cluttered maritime background. The only other publicly available database of which we are aware contains aligned dynamic Visual and LWIR imagery representing driving scenarios in urban environments. It is intended for the development of visual place recognition algorithms, and not suited for the development of image fusion algorithms [77].

We provide the TRICLOBS image data set (described in this paper) to fill this data gap. This data set contains registered three-band dynamic imagery of different surveillance scenarios showing various types of human activity. This imagery can be used for the development and evaluation of (both static and dynamic) image fusion, enhancement and color mapping algorithms. The content outline for the rest of this paper is as follows. First, we describe the TRICLOBS camera system that was used to collect the imagery and the locations and scenarios that were registered. Next, we describe the structure and content of the TRICLOBS data set. Then, we present some sample applications that use the TRICLOBS imagery as input. Finally, we end with some concluding remarks and note some limitations of the data set.

## Materials and Methods

### The TRICLOBS multi-band camera system

We recently developed the TRICLOBS (TRI-band Color Low-light OBServation) all-day all-weather surveillance and navigation system which contains three optically aligned cameras that are sensitive to the Visual, NIR and LWIR parts of the electromagnetic spectrum [78]. The system combines (fuses) the three camera signals in real-time in a false-color RGB signal. It has the capability to perform color remapping using either pre-recorded lookup tables [79] or by deriving the color mapping in real-time from synthetic imagery or Google Earth data [80]. As a result the system provides dynamic imagery with a realistic color appearance in conditions of low visibility (low light levels, smoke). A recent evaluation study using static imagery has shown that human scene inspection and recognition performance with color remapped TRICLOBS imagery resembles the performance with standard color photographs [5].

The TRICLOBS can also be extended with a synthetic 3D scene generation system in combination with an additional image fusion and image processing module. The result is INVIS, an Integrated Night VIsion surveillance and observation System: [81]. For an online demonstration of INVIS’ capabilities, see [82, 83]).

This section describes the TRICLOBS system and the image registration procedure. In addition, we briefly explain the color remapping procedure that was used to create the false-color movies thus illustrating the contents of this data set. More details of the hard- and software of the TRICLOBS system [78] and the color mapping procedure [79] are presented elsewhere.

### Sensor suite

**Fig 1:**
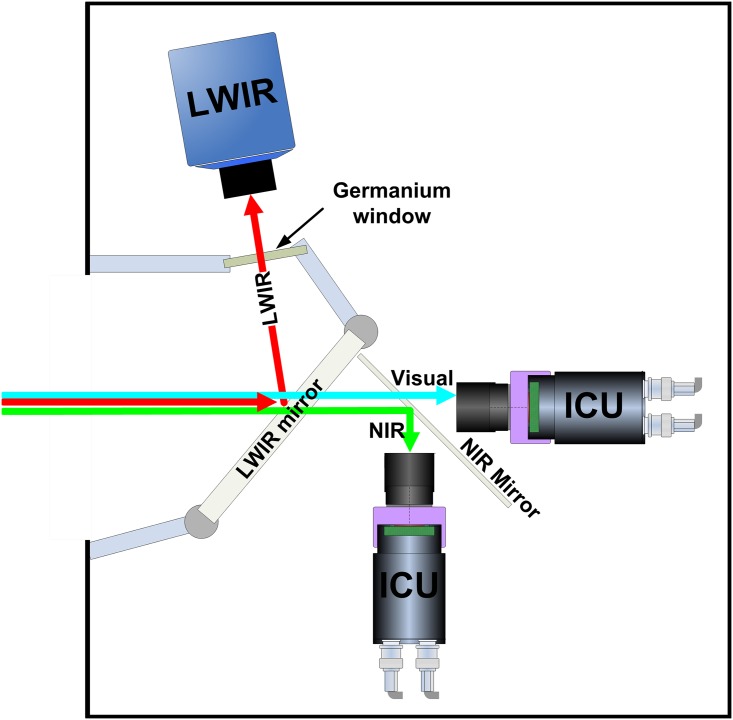
Schematic representation of theTRICLOBS sensor suite layout. The long-wave part of the incoming radiation is reflected into an uncooled infrared microbolometer by a LWIR (hot) mirror, the near-infrared part is reflected by a dichroic beam splitter (NIR mirror) towards an image intensifier (ICU), while the visual part goes straight to a second image intensifier.

Fig 1 shows a schematic representation of the layout of the TRICLOBS sensor suite and its optical components. The system contains two digital image intensifiers and a thermal camera.

The two image intensifiers are high resolution (1280 × 960) Photonis PP3000U Intensified Camera Units (ICU’s, www.photonis.com). The ICU is a low-light-level, intensified CMOS camera. It has a 16.9mm (2/3") CMOS sensor with a spectral response range of 0.4–0.9μm. It delivers an SDI—LVDS 270 Mbits/s signal and a PAL or NTSC composite video signal outputs (ITU-R BT.656-4, 640 × 480 pixels, 25 frames/s). Both ICU’s are equipped with Pentax C2514M CCTV lenses, with a minimal focal length of 25mm and a lens aperture of F/1.4, resulting in a 30.7° × 24.8° field-of-view (FOV.)

The thermal camera is a XenICs Gobi 384 uncooled a-Si infrared microbolometer (www.xenics.com). It has a 384 × 288 pixel focal-plane array, and a spectral sensitivity range of 8–14μm. This is the range of most interest for outdoor surveillance applications. The camera is equipped with an Ophir supIR18mm F/1 lens (www.ophiropt.com) providing a 29.9° × 22.6° FOV. The Gobi 384 has 16-bit Ethernet and CameraLink interfaces running at 44 frames/s.

**Fig 2:**
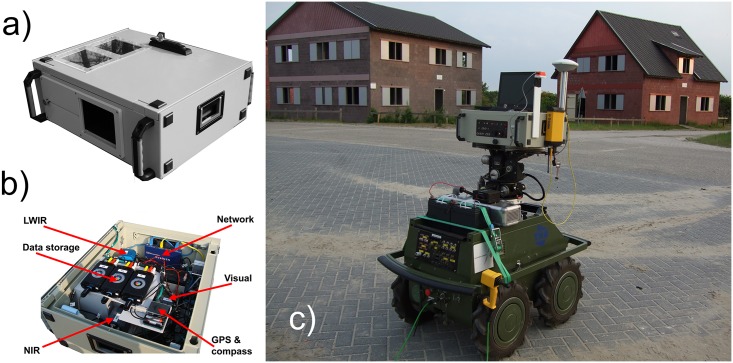
The TRICLOBS system. (a) The system is contained in a water and dust resistant housing with a single aperture and built-in displays that enable signal monitoring. (b) The interior showing the sensors and other components. (c) The TRICLOBS system mounted on an all-terrain platform.

Two beam splitters are deployed to direct the appropriate band of the incoming radiation to each of the three individual sensors (Fig 1). The incoming radiation is first split into a (thermal) long-wave infrared (LWIR) part and a Visual+NIR part by a heat reflecting (hot) mirror. The hot mirror is a custom made Melles Griot dichroic beam splitter consisting of Schott N-BK7 Borosilicate Crown glass with an Indium Tin Oxide coating. It has a reflection coefficient of R > 85%. The LWIR part of the spectrum is reflected into the lens of the thermal camera, while the Visual+NIR light is transmitted to a combination of two digital image intensifiers that are mounted at an angle of 90 degrees. Next, an NIR reflecting mirror with 45 deg angle of incidence, Borofloat glass, and type Edmund Optics B43-958, 101 × 127 × 3.3 mm (see: www.edmundoptics.com separates the incoming light by transmitting the Visual (0.4–0.7μm) and reflecting the NIR part (0.7–0.9μm), in such a way that one image intensifier registers the visual part and the other one only detects the NIR part of the incoming radiation. The sensor geometry is such that the optical axes of all cameras are aligned. The sensors and the mirrors are mounted on a common metal frame. The whole configuration is portable and contained in a dust and water resistant housing (Fig 2a and 2b) that could be mounted easily onto a mobile platform (Fig 2c). A Germanium window covers the aperture of the thermal camera. The sensor suite delivers both analog video and digital signal outputs.

### GPS receivers

An internal U-blox EVK-5P Positioning Engine (www.u-blox.com) provides a position and orientation (i.e., sensor location and viewing direction) signal through the high-speed 7-port USB 2.0 hub. The accuracy in position is less than 3m. The accuracy in orientation is less than 5 degrees. In local area operations when high accuracy is required, an external Trimble SPS751 GPS receiver set (www.trimble.com) is connected to the system, to achieve high position accuracy (< 1 cm) through real time kinematic (RTK) GPS signal correction. The position information provided by the U-blox Positioning Engine can be used to load color look-up tables (see the section on color remapping) that are optimal for the environment in which the TRICLOBS system is being deployed [80–83].

### Electronic compasses

An internal Silicon Labs F350-COMPASS-RD multi-axis electronic compass (www.silabs.com) provides the azimuth and tilt angle of the optical axis of the sensor suite with an accuracy of a few degrees (Fig 2b). When the viewing direction needs to be known with higher accuracy, an external Xsens 3D inertial measurement unit (IMU) motion sensor with an accelerometer, magnetometer and gyroscope (www.xsens.com) is connected to the system to measure Yaw, Roll en Pitch with an accuracy less than 0.1°. The viewing direction provided by the electronic compass can for instance be used to render a view from a synthetic geometric 3D scene model that corresponds to the viewing direction and viewpoint of the TRICLOBS camera system [80–83].

### Computer

A Dell Precision M2400 Intel Core Duo P8600 2.4GHz laptop with a solid state hard disk is used to store, colorize, and visualize the sensor signals and to generate and display the synthetic scene view. The current implementation achieves real-time (~25 Hz) visualization, signal enhancement and data registration.

### Displays

Two 6.4” TFT video displays, embedded in the system casing, enable simultaneous monitoring of two of the three video signals (either Visual/NIR, Visual/LWIR, or NIR/LWIR; Fig 2a). The laptop display (14 inch, 1440x900 pixels) is used to view the final fused, colored and enhanced images.

### Data transfer and storage

The Photonis ICU’s are connected to a high-speed 7-port USB 2.0 hub. This enables the user to interface with the ICU’s and to adjust their settings, or to download and install preferred settings.

A Pleora iPORT PT1000-ANL-2/6 frame grabber (www.pleora.com) digitizes the analog video output signals of (1) both ICU’s and (2) the Gobi 384. Digitization is performed at a rate of 25 frames/s with a resolution of 640x480 pixels and 10 bits per pixel.

The three sensors are not frame synchronized. Instead, the images of each of the three sensors are stored with a time stamp (in ms). A three band image sequence is then constructed by combining each Visual frame with the NIR and LWIR frames with nearest time stamps in a single RGB frame. This procedure results in an average temporal offset between the Visual and NIR bands of 7.5±1 ms and an average temporal offset between the Visual and IR bands of 9±1 ms. Thus, the temporal offset between the individual bands was less than one frame.

The Pleora transmits these signals to a Netgear Gigabit Ethernet switch. The 16-bit TCP/IP Ethernet interface of the XenICs Gobi 384 is also directly connected to the Netgear Gigabit Ethernet switch.

Three Pinnacle Video Transfer Units (www.pinnaclesys.com/PVT) store (a) the analog video signals of all three cameras, and (b) the audio signals of two (optional) external microphones, either on 3 internal 320 Gb hard disks, or on USB memory sticks. The microphones can be positioned on the front and back of the camera suite. The microphone on front could then be used to register relevant audio information from the registered scene, and the second microphone could be used to record spoken annotations.

### Image registration

The Visual (0.4–0.7μm) and NIR (0.7–0.9μm) images provided by the two ICU digital image intensifiers have a size of 640 × 480 pixels and represent a FOV of 30.7° × 24.8°. The LWIR (8–14μm) image provided by the XenICs Gobi 384 thermal camera has a size of 384 × 288 pixels and represents a FOV of 29.9° × 22.6°. As a result of the optical alignment of the camera systems, the FOV of the LWIR image corresponds to the central part of the FOV of the Visual and NIR images. The size of this common FOV area is 621 × 461 pixels in the Visual and NIR images. To enable the fusion of the LWIR image with the other two channels, the LWIR image (384 × 288 pixels) is bi-linearly interpolated and up-sampled (by a factor of about 1.6) to 621 × 461 pixels. The Visual and NIR images are cropped to their central part of 621 × 461 pixels so that only the common FOV area remains. Finally, all images are rescaled to 640 × 480 pixels. This is also the size of the three-band color images provided in the database presented in this study. As a result, an individual pixel represents about 2.8 × 2.8 min of arc of the visual field.

### Color remapping

The TRICLOBS system has the option to deploy a recently developed color remapping technique [79]. This mapping assumes a fixed relation between false-color tuples and natural color triplets for bands near the visual spectrum. This allows its implementation as a simple color table swapping operation. For bands that are not correlated with the visual spectrum (such as LWIR) this assumption evidently does not apply. In that case, the color remapping could be used to improve the detectability of targets through contrast enhancement and color highlighting [84].

**Fig 3:**
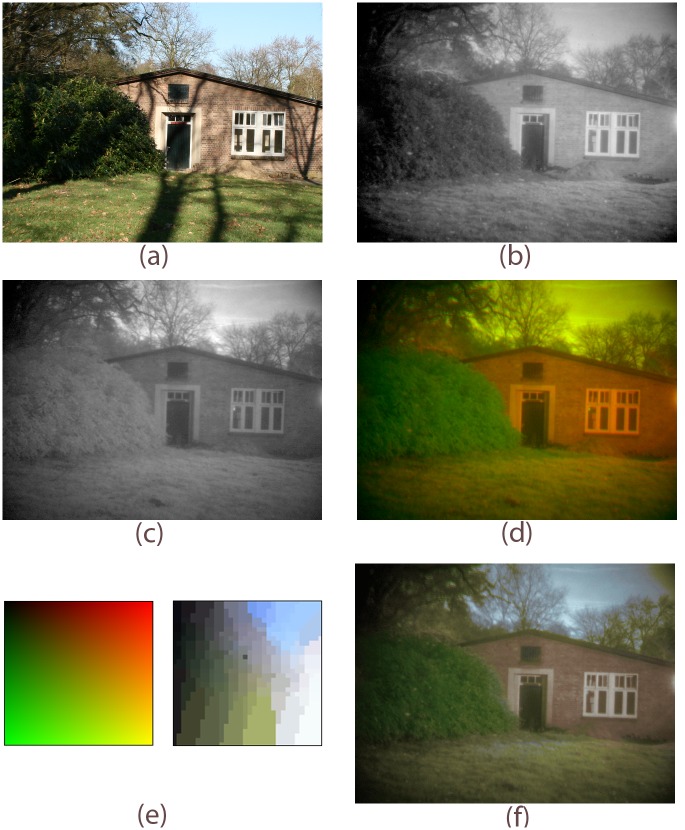
Color remapping procedure. (a) Daylight color reference image. Visible (b) and NIR (c) images of the same scene as in (a). (d) Two-band (RG) false-color image obtained by assigning (b) to the green and (c) to the red channel of an RGB color image. The blue channel is set to zero. (e) The color mapping derived from corresponding pixel pairs in (a) and (d). (f) Result of the application of the mapping scheme in (d) to the false-color image in (d).

Color remapping can be achieved by mapping the multi-band sensor signal to an indexed false-color image and swapping its color table with that of a regular daylight color image of a similar scene (see Fig 3). Different (e.g., urban, rural, maritime, or desert) environments may require specific color tables. However, in practice we found that an entire environment is well represented by a single color table, as long as the environmental characteristics don’t change too drastically [85]. Thus, only a limited number of color tables is required in practice. These tables need to be constructed only once, before the system is deployed.

For a given environment, the lookup color table transformation can be derived as follows. First, take a multispectral image of a scene that is typical for the intended operating theater and transform this image to an indexed color representation. Second, take an indexed representation of a regular color photograph of a similar scene. Then there are two options [86]. Either transfer the first order statistics of the lookup table of the color photograph to the lookup table of the false-color multispectral image [68], or establish a direct mapping between corresponding entries in both tables [79].

When matching multispectral and daylight color images of the same scene are available, a realistic color mapping can be obtained by establishing a direct relation between the values of corresponding pixels in both images [79]. When there is only a daylight color image available of an environment that is similar to the one in which the multispectral sensor suite will be deployed, a mapping can still be established by transferring the color statistics of the daylight image to the multispectral image [68]. Although the first approach yields more specific colors, both approaches produce intuitively correct and stable color representations. Note that the statistical approach can even be used with imagery from sources like artificial terrain databases or Google Earth [80] (for demonstrations see [82] and [83]). The specificity of the lookup-table color remapping has the additional advantage that it enables to selectively enhance and emphasize details of interest (e.g., camouflaged targets) in a given scene [79, 87, 88].

For the sake of completeness we will briefly describe our color transformation here, using the example shown in Fig 3. (A full description of the method is presented elsewhere [79]). Fig 3a depicts the full color daytime reference image, which is a color photograph taken with a standard digital camera. Fig 3b and 3c show a visible and near-infrared image of the same scene. Fig 3f shows the result of applying daytime colors to the two-band night-time sensor image using our new color mapping technique. The color transfer method works as follows. First, the three-band sensor image is transformed to a false-color RGB image by taking the individual Visual (Fig 3b), NIR (Fig 3c), and LWIR bands as input to the R,G and B color channels respectively. The result is a false-color RGB-image (Fig 3d). In this example we assume that the LWIR signal is absent (black) and we consider only the Visual and NIR (R and G) channels, because these two channels are typically used to produce naturalistic colors while the LWIR (B) channel usually serves to enhance target distinctness. Note that the actual order of the initial mapping of the sensor channels to the RGB channels is irrelevant. Mapping the sensor bands to a false-color RGB-image allows us to use standard image conversion techniques, such as indexing [89]. In the next step the resulting false-color (RGB) image (Fig 3d) is converted to an indexed image. Each pixel in such an image contains a single index. The index refers to an RGB-value in a color look-up table. The number of entries can be chosen by the user. In the present example of a sensor image consisting of two bands (R and G; Fig 3d) the color look-up table contains various combinations of R and G values. Here the B-values are ignored since only the Visual and NIR bands are considered, however the procedure is identical when all three channels are considered. For each index representing a given R,G combination (i.e., for a given false color) the corresponding realistic color equivalent is obtained by locating the pixels in the target image with the same index and collecting the corresponding pixels in the (realistic color) reference image (Fig 3a). First, the RGB-values are converted to perceptually de-correlated lαβ values [90]. Next, the average lαβ-vector is calculated over this ensemble of pixels. Averaging guarantees that the computed average color reflects the perceptual average color. Averaging automatically takes the distribution of the pixels into account. Colors that appear more frequently are given a greater weight. For instance, let us assume that we would like to derive the realistic color associated with color index *i*. In that case we locate all pixels in the (indexed) false-color multi-band target image with color index *i*. We then collect all corresponding pixels (i.e., pixels with the same image coordinates) in the reference daytime color image, convert these to lαβ, and calculate the average lαβ-value of this set. Next, we transform the resulting average lαβ-value back to RGB. Finally, we assign this RGB-value to index *i* of the new color look-up table. These steps are successively carried out for all color indices. This process yields a new color look-up table containing the realistic colors associated with the various multi-band combinations in the false-color (RGB) look-up table. Replacing the RGB-color look-up table (left side of Fig 3e) by the realistic color look-up table (right side of Fig 3e) yields an image with a realistic color appearance, in which the colors are optimized for this particular sample set (Fig 3f).

### Registration sites and conditions

The imagery in the TRICLOBS data set were collected at three different locations and show three different scenes.

The first part of the imagery (TRI_A) was collected at Marnehuizen, The Netherlands (53.386311 deg North latitude, 6.262761 deg East longitude) (see https://nl.wikipedia.org/wiki/Marnehuizen). Marnehuizen is a Dutch mock-up village built to train soldiers and police for operations in urban environments. Marnehuizen consists of houses, sheds, shops, a bank, a school, a town hall, a gas station and a village square. Street furniture like lights, telephone booths, shrubs and trees, street signs, fences, parking lots, and car wrecks give the village a realistic appearance.

The second part of the imagery (TRI_B) was registered at the training grounds of the Royal Netherlands Army Camouflage School at Reek, The Netherlands (51.729450 deg North latitude, 5.705908 deg East longitude). This site also contains a few realistic houses with some street furniture for training purposes.

The third part of the imagery (TRI_C) was collected at the site of the TNO laboratories in Soesterberg, The Netherlands (52.115469 deg North latitude, 5.290877 deg East longitude). The imagery was collected near a side entrance of the main building, where the walls were covered with foliage and a path leads to glass doors.

An ‘additional_data’ folder in the TRICLOBS data set contains Excel and CSV files that present information on the environmental (atmospheric and lighting) conditions during the actual image registration periods.

### Scenarios

Table 1 gives a brief description of the different scenarios represented in the TRICLOBS dynamic image data set. The scenes that are registered are representative for typical short-range (military or civilian) surveillance scenarios. They include people, vehicles, foliage and buildings or other man-made structures. The people are either stationary, walking or running and dressed in military or in civilian clothing. They perform a range of different (sometimes suspicious) activities, such as carrying different objects (box, gun, axe, stick), loitering, hiding in the foliage, inspecting and entering buildings, fighting, walking around and driving vehicles. Their image may vary from completely unobstructed, to partially occluded and fully occluded during the course of a scene. The imagery was collected for a range of different lighting conditions, ranging from just before (Scenario C) and just after (Scenario B) sunset to full darkness (Scenario A). The range was chosen so as to ensure a variation in the information content in the different spectral bands. Most scenarios present outdoor human activities, while some also show activities inside buildings (Scenarios B1-3, C3-4). Some scenes (Scenarios A3, C2) contain smoke that obscures the Visual and NIR channels. Note that scenes in which people are behind smoke (opaque for Visual and NIR) or glass (opaque for LWIR) yield imagery with complementary information content because the different spectral bands represent different details in the scene. The scenarios are particularly useful for the evaluation of image fusion algorithms.

**Table 1:** Brief description of the scenarios represented in the TRICLOBS database.

Scenario	No. of Frames	Action
A1	687	Three soldiers park a jeep on a small village square surrounded by a brick wall, descend from the vehicle and walk away.
A2	655	The camera pans over a scene with houses, trees and semi shrubs and an abandoned car wreck.
A3	1744	The scene represents a path between two houses. Smoke from a grenade that is thrown into the scene fills the space between the two houses. An armed soldier appears from behind the house on the left, crawls over the ground and enters the house on the right. The soldier leaves the house and walks away.
A4	1573	The camera pans back and forth over a scene with a row of houses and a bank. There is a roll of barbed wire on the ground in front of the bank. A person runs from right to left and disappears behind the house next to the bank. An armed soldier leaves the bank.
B1	1325	The scene shows the facade of a building with glass doors and foliage in front of the walls. A person comes out of the foliage on the right, hides in the foliage on the left, enters the building, leaves the building with a large box, and hides in the foliage.
B2	5910	Same scene as in B1. A person emerges from the foliage on the left, enters the building, waits until other people have left the building, opens the door and inspects the entrance with a flashlight, enters the building again, walks back and forth inside the hallway, leaves the building and walks back and forth between the foliage on both sides of the pathway.
B3	1517	Same scene as in B1. A person emerges from the foliage on the left, enters the building, leaves the building with a large box while carrying a flashlight, and hides in the foliage.
B4	2574	Two persons, one carrying a stick and the other one carrying a small axe, pass behind a glass shelter, enter the scene from behind the shelter, start a fight using their axe and stick, and walk away.
C1	6133	The scene shows the facades of two houses with a lawn in the foreground. In front of the house on the left there is a hedge. A small hot object lies on the ground at the right end of the hedge. A person enters the scene from left, disappears behind the hedge, returns with a box, drops the box over the hot object on the ground, and leaves the scene on the left. Somewhat later the person returns, removes the box from the hot object, drops it behind the hedge, and leaves the scene again. Later, armed soldiers and a civilian walk through the scene from left to right, passing by the hot object on the ground.
C2	7257	Same scene as in C1. Smoke from a grenade thrown on the middle of the lawn gradually obscures parts of the scene. Soldiers and civilians walk through the smoke.
C3	3843	Same scene as in C1. Persons move behind the open (upper left) and closed (upper right) windows of the house on the left. A person opens and closes the upper left window of the house on the right. Two armed soldiers walk along the path between the two houses.
C4	3695	Same scene as in C1. Two armed soldiers inspect the house on the left. One inspects the upper floor and looks out of the open window on the left. The other leaves the building through the backdoor and appears on the right side of the house on the left. Persons move behind the upper left window and in the doorway of the house on the right. The two soldiers walk past the house on the right an leave the scene.
C5	10365	Same scene as in C1. Two persons with a jeep repeatedly drive along to deliver goods: first two times to the house on the right, then twice to the house on the left.
C6	6657	Same scene as in C1. A person enters the scene from left, disappears behind the hedge, returns with a box, drops the box over the hot object on the ground, and leaves the scene on the left. Civilians carrying a range of different objects (e.g., a briefcase, a rake) and soldiers (some carrying guns) walk through the scene past the hot object.
C7	901	Same scene as in C1. Two civilians walk through the scene carrying an elongated object.
C8	2029	Same scene as in C1. Two armed soldiers on patrol walk past the hot object on the ground.

The only individuals that appear in this manuscript and in the TRICLOBS dynamic image data set are the authors of this paper. These individuals have given written informed consent (as outlined in PLOS consent form) to publish these case details.

### The TRICLOBS Data Set: Contents and Structure

The TRICLOBS image database (publicly available from https://figshare.com/articles/The_TRICLOBS_Dynamic_Multiband_Image_Dataset/3206887 with DOI 10.6084/m9.figshare.3206887) consists of three parts: TRI_A, TRI_B, and TRI_C, with imagery that was collected at sites in Marnehuizen, Reek, and Soesterberg (The Netherlands), respectively.

The main folders TRI_A, TRI_B, and TRI_C contain 4, 4, and 8 subfolders, respectively. Each subfolder contains TRICLOBS imagery of the different scenarios registered at each of the three different locations. In addition, each of the folders TRI_A, TRI_B, and TRI_C also contains a folder with the extension ‘photographs’, which contains full-color photographs of the registration site. These photographs can be used to derive color mappings that give the TRICLOBS multi-band imagery a realistic color appearance.

The subfolders for the individual scenarios each contain a folder with the extension ‘frames’ and a movie in MP4 format.

**Fig 4:**
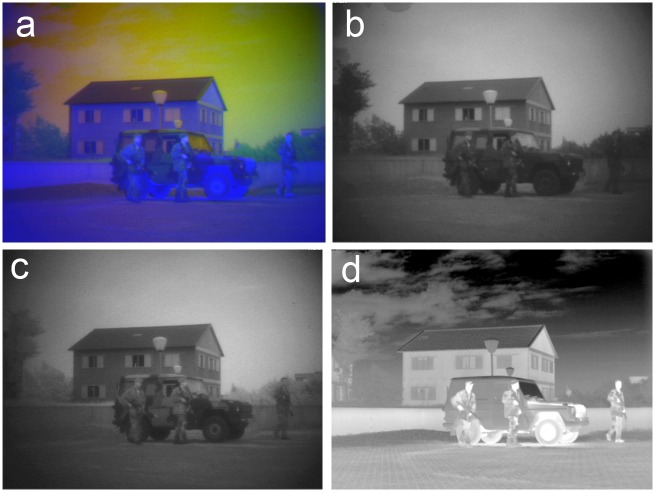
Example RGB false-color frame and its individual channels (from scenario TRI_A1). False-color RGB frames in the TRICLOBS database (a) are constructed by mapping corresponding (b) Visual frames to the Red channel, (c) NIR frames to the Green channel and (d) LWIR frames to the Blue channel of an RGB color image.

The ‘frames’ folders contain sequentially numbered false-color RGB images in 24-bit BMP format and with a size of 640 × 480 pixels (e.g., Fig 4). Each of these RGB images is composed of three corresponding (i.e., nearly simultaneously grabbed) 8-bit frames from the Visual (R), NIR (G), and LWIR (B) channels, respectively, of the TRICLOBS system. Since all frames are sequentially numbered the user may use any video capture and processing tool to view them as a video stream. An example of an efficient tool for this purpose is VirtualDub, which is licensed under the GNU General Public License and is freely available from http://www.virtualdub.org. With VirtualDub the images in a given frames folder can simply be inspected as a video stream by using the ‘File > Open video file’ option and selecting the first image in the frames folder. VirtualDub will then automatically open the entire range of sequentially numbered frames and a slider below the image display allows easy navigation through the motion sequence.

**Fig 5:**
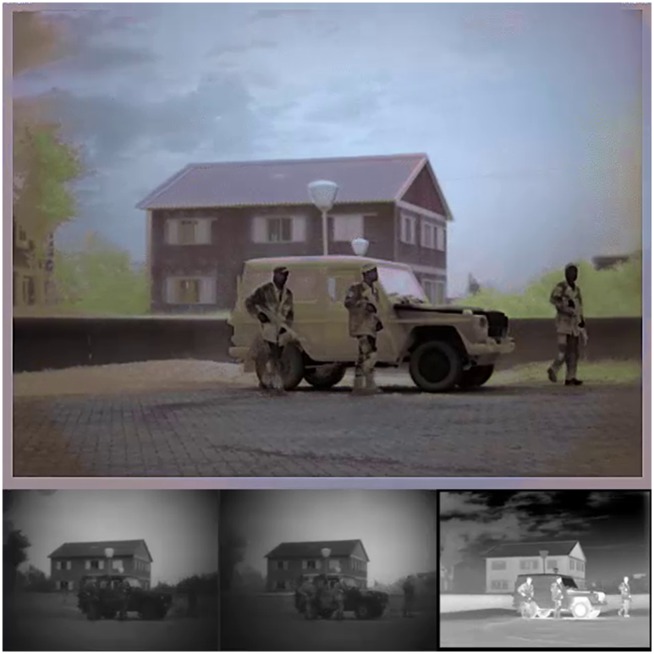
Example MP4 movie frame (from TRI_A1.mp4). The MP4 movies consist of four panels: the lower three panels represent the Visual (left), NIR (middle) and LWIR (right) channels, while the upper panel shows the fused result after color remapping performed on the TRICLOBS system.

The MP4 movies consist of four panels: the lower three panels represent the Visual (left), NIR (middle) and LWIR (right) channels, while the upper panel shows the fused result after color remapping on the TRICLOBS system (e.g., Fig 5). These movies only serve to give the user a quick impression of the contents of the corresponding frames folders (by dynamically showing the individual channels in the lower three panels) and the effects that may be achieved by realistic color remapping of the false-color frames (upper panel). They are not intended for further processing.

## Example Applications

### Image fusion

**Fig 6:**
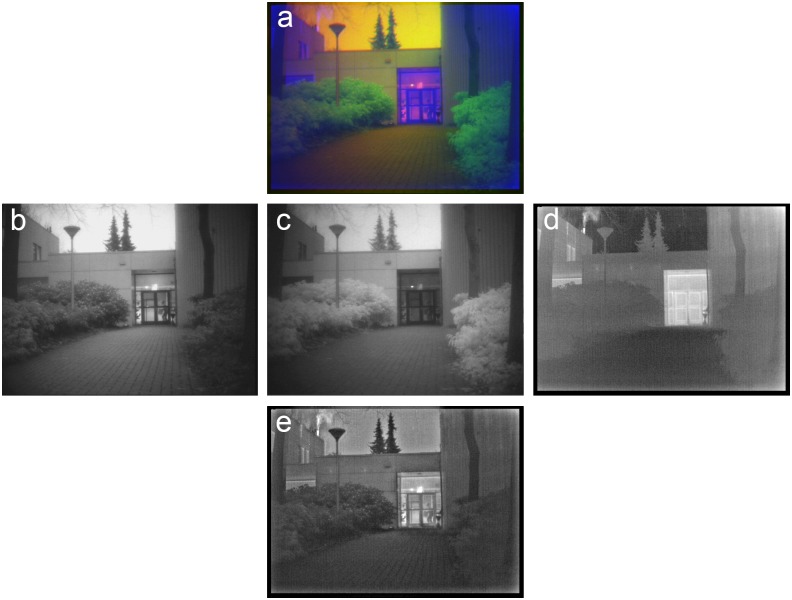
Example of grayscale image fusion. (a) False-color frame from series TRI_B1, with the Visual (R) channel (b), the NIR(G) channel (c) and the LWIR (B) channel (d). (e) Result of grayscale (Laplacian pyramid) fusion of (b-d). The person carrying a box behind the glass door is quite distinct in the Visual band but not represented in the LWIR band. In contrast, smoke from the chimney on the upper left is quite distinct in the LWIR band but not represented in the other bands.

**Fig 7:**
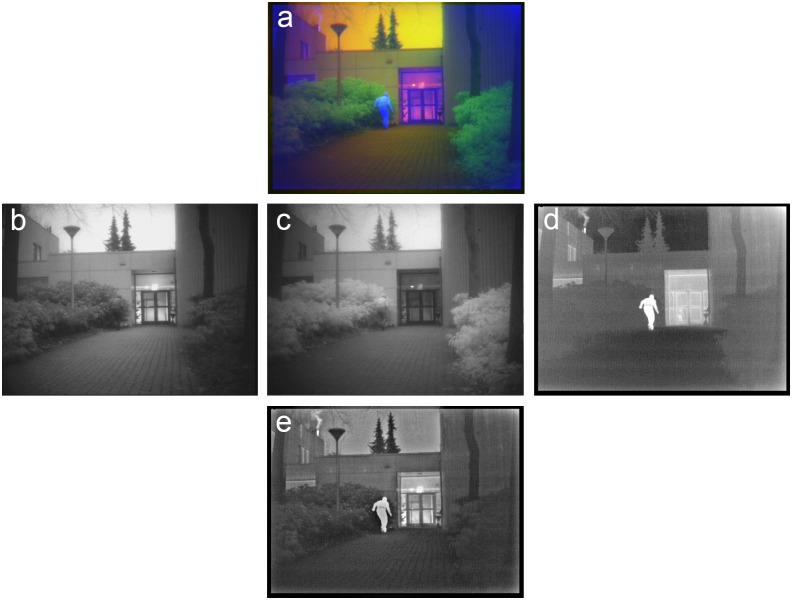
Example of grayscale image fusion. (a) False-color frame from series TRI_B2 with the Visual (R) channel (b), the NIR(G) channel (c) and the LWIR (B) channel (d). (e) Result of grayscale (Laplacian pyramid) fusion of (b-d). The person emerging from the foliage and the smoke plume rising from the chimney on the left are both highly visible in the LWIR band (d) but hard to distinguish in the other two bands. Both these details are represented with high contrast in the grayscale fused image (e) that is obtained through Laplacian pyramid fusion of the three individual bands.

**Fig 8:**
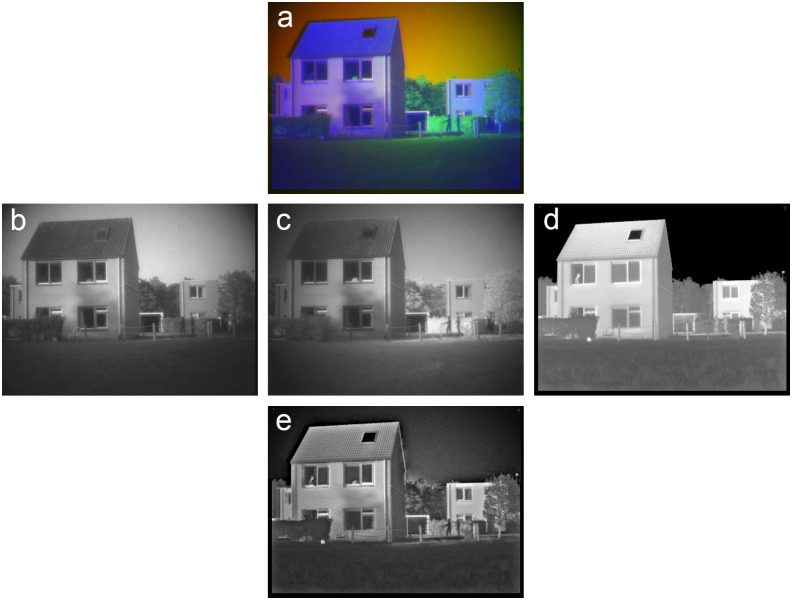
Example of grayscale image fusion. (a) False-color frame from series TRI_C3 with the Visual (R) channel (b), the NIR(G) channel (c) and the LWIR (B) channel (d). (e) Result of grayscale (Laplacian pyramid) fusion of (b-d). The persons behind the two upper windows in the house on the foreground (left) and the two soldiers in front of the hedge in the back are visible in different bands but are all represented in the fused image (e).

Figs 6–8 illustrate the use of individual false-color RGB frames for testing image fusion algorithms. The grayscale image fusion method used in these examples is a 4 layer Laplacian pyramid [91], using simple averaging to compute the lowest resolution level of the fused image representation [92].

Fig 6a shows a frame from the TRI_B1 sequence in the TRICLOBS database. This scene represents a building with a glass door and a person carrying a box behind the door. There is foliage on both sides of the path leading to the door. Smoke rises from a chimney on the left. Notice that there is a person behind the glass door carrying a large box. This person is quite distinct in the Visual band (Fig 6b), much less visible in the NIR band (Fig 6c) and invisible in the LWIR band (Fig 6d) because glass is opaque to thermal radiation. The smoke from the chimney on the roof of the building on the left is clearly visible in the LWIR band (thermal contrast) but not represented in the other two bands. Note that all these details are clearly represented in the grayscale fused image that is obtained by fusion of the three individual bands with a Laplacian pyramid algorithm (Fig 6e).

Fig 7a shows a frame from the TRI_B2 sequence in the TRICLOBS database. This scene is similar to the scene in Fig 6 except for the person, who now emerges from the foliage on the left side of the pathway. His light jacket and dark trousers blend well into the light foliage and dark shadows beneath the semi-shrubs with virtually no luminance contrast. Similarly, the smoke rising from the chimney on the left has no luminance contrast with the sky. As a result both details are hard to distinguish in the Visual (Fig 7b) and NIR (Fig 7c) bands. However, they are both highly visible in the LWIR band (Fig 7d) due to their temperature contrast. Both these details are represented with high contrast in the grayscale fused image (Fig 7e) that is obtained by fusion of the three individual bands with a Laplacian pyramid algorithm.

Fig 8a shows a frame from the TRI_C3 sequence in the TRICLOBS database. This scene shows a house in the foreground with a slanting roof with a shed attached and a house with a flat roof in the background. Note the two upper windows in the house on the foreground (left). The left upper window is open while the right one is closed. The person behind the open window is only visible in the LWIR band (Fig 8d: high thermal contrast), while the person behind the closed window can only be seen in the NIR band (Fig 8c) since glass is opaque to LWIR. The two soldiers walking in front of the hedge in the background are most distinct in the Visible (Fig 8b) and in the NIR (Fig 8c) band. Note that all persons are clearly visible in the grayscale fused image (Fig 8e). This image results from fusion of the three individual bands with a Laplacian pyramid algorithm.

### Color remapping

**Fig 9:**
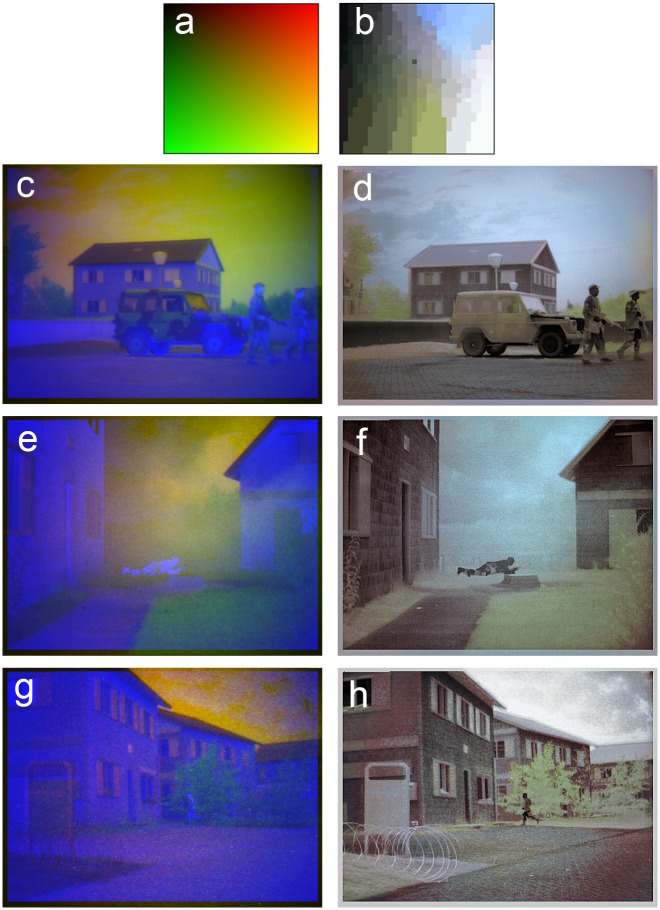
Color remapping applied to false-color RGB frames from the TRICLOBS data set. Applying a color remapping (defined by the color table pair a,b) to RGB false-color frames (c, e and g) gives them a realistic color appearance (d, f and h).

Fig 9 presents some examples of the application of color remapping to raw RGB false-color frames from the TRICLOBS image data set. The transformation is defined by the lookup table pair shown in Fig 9a and 9b, and was performed according to the procedure described previously in the Materials and Methods section (for a full description of the method see [79]). The color table shown in Fig 9a represents all possible RG combinations that can appear in a RGB color image. The B or LWIR channels is not considered here since it has no relation with natural colors. The color table shown in Fig 9b was generated by relating all the RG tuples to corresponding RGB triples in a color photographs matching a TRICLOBS false-color image. This mapping gives the false-color frames a realistic color appearance, as shown in Fig 6d, 6f and 6h. In a previous study we found that this type of color-remapped multi-band image significantly enhanced human perception. With color remapped imagery, observers correctly perceive more details (i.e., they can extract the gist) of a scene in a single glimpse compared to conventional monochrome image representations [5].

## Concluding Remarks

In this paper we presented the TRICLOBS image data set which is the first publicly available dataset that provides registered Visual, NIR and LWIR dynamic image sequences representing different surveillance scenarios. The imagery was collected during several nightly field trials using our newly developed TRICLOBS camera system. The resulting data set is intended for the development and evaluation of image fusion, enhancement and color mapping algorithms for military and civilian short-range surveillance scenarios. Individual frames from the data set contain registered Visual, NIR and LWIR images. These can be used to develop and test static image fusion and color remapping algorithms. Sequences of frames can be used for the development of dynamic image fusion algorithms. TRICLOBS imagery has been used successfully in previous studies to develop new color mapping schemes to give multi-band night vision imagery a realistic color appearance [73, 86], to design new image fusion schemes [63], to assess the added value of color fused image representations for human observation [5], to construct an augmented reality nighttime surveillance system [80], and to evaluate a synthetic observer approach to multisensory resolution assessment [93, 94].

### Limitations of the data set

The TRICLOBS image data set also has some limitations.

Currently only a limited number of scenarios and scenes are included. To increase the value of the data set we defined scenarios that are generic for (military and civilian) surveillance applications, including a wide range of different objects (white hot targets, weapons, briefcase, vehicles, etc.). Also, the range of atmospherics and lighting conditions for which imagery was registered is rather limited, ranging from just before, just after, to long after sunset. In the future, we plan to extend the data set with imagery of other natural, as well as urban, environments, registered for a wide range of atmospheric conditions.

The digitization of the three TRICLOBS sensor channels was not synchronized. However, the maximal time delays were quite small (< 9 ms). As a result, registration errors may only become visible when objects move through the scene at high speed.

The two Photonis ICUs had independent automatic gain regulation, which resulted in some minor flicker effects in some conditions. The user can correct these effects by normalizing both Visual and NIR image sequences.

The images of the two Photonis ICUs show some vignetting (a reduction of the image brightness towards the edges). The user can either correct this effect by applying a digital contrast enhancement procedure or by using only the central part of the frames for image fusion applications.

## References

[1] Blum RS, Liu Z. Multi-sensor image fusion and its applications. Boca Raton, Florida, USA: CRC Press, Taylor & Francis Group; 2006.

[2] Gade R, Moeslund TB. Thermal cameras and applications: a survey. Machine Vision and Applications. 2014;25(1):245–62.

[3] Toet A, IJspeert JK, Waxman AM, Aguilar M. Fusion of visible and thermal imagery improves situational awareness. Displays. 1997;18(2):85–95.

[4] Toet A, Franken EM. Perceptual evaluation of different image fusion schemes. Displays. 2003;24(1):25–37.

[5] Toet A, de Jong MJ, Hogervorst MA, Hooge ITC. Perceptual evaluation of color transformed multispectral imagery. Optical Engineering. 2014;53(4):043101–12.

[6] Cohen N, Mizrahni G, Sarusi G, Sa'ar A. Integrated HBT/QWIP structure for dual color imaging. Infrared Physics & Technology. 2005;47(1–2):43–52.

[7] Vandersmissen R. Night-vision camera combines thermal and low-light level images. Photonik international. 2008;2008(2):2–4.

[8] Bandara SV, Gunapala SD, Liu JK, Rafoi SB, Ting DZ, Mumulo JM, et al Four-band quantum well infrared photodetector array. Infrared Physics & Technology. 2003;44(5–6):369–75.

[9] Cho E, McQuiston BK, Lim W, Gunapala SD, O'Neill J, Hutchinson A. Development of a QWIP dual-color FPA for mine detection applications. In: Andresen BF, Fulop GF, editors.;Vol SPIE-5074; Bellingham,WA: The International Society for Optical Engineering; 2003 p. 685–95.

[10] Goldberg AC, Fischer T, Derzko Z, Uppal PN, Winn M. Development of a dual-band LWIR/LWIR QWIP focal plane array for detection of buried land mines In: Dereniak EL, Sampson RE, editors. Infrared Detectors and Focal Plane Arrays VII;Vol SPIE-4721; Bellingham, WA: The International Society for Optical Engineering; 2002 p. 184–95.

[11] Goldberg AC, Uppal P, Winn M. Detection of buried land mines using a dual-band LWIR/LWIR QWIP focal plane array. Infrared Physics & Technology. 2003;44(5–6):427–37.

[12] Goldberg AC, Stann AC, Gupta N. Multispectral, hyperspectral, and three-dimensional imaging research at the U.S. Army Research Laboratory. Sixth International Conference on Information Fusion (FUSION 2003); Fairborn, OH, USA: International Society of Information Fusion; 2003. p. 499–506.

[13] Goldberg AC, Fischer T, Derzko ZI. Application of dual-band infrared focal plane arrays to tactical and strategic military problems In: Andresen B, Fulop GF, Strojnik M, editors. Infrared Technology and Applications XXVIII;Vol SPIE-4820 Bellingham, WA., USA: The International Society for Optical Engineering; 2003 p. 500–14.

[14] Kriesel JM, Gat N. True-color night vision (TCNV) fusion system using a VNIR EMCCD and a LWIR microbolometer camera In: Kadar I, editor. Signal Processing, Sensor Fusion, and Target Recognition XIX;Vol SPIE-7697; Bellingham, WA, USA: The International Society for Optical Engineering; 2010 p. 76970Z 1–8.

[15] Ma W, Wang S, Wen Y, Zhao Y, Dong L, Liu M, et al. Uncooled multi-band IR imaging using bimaterial cantilever FPA. IEEE 27th International Conference on Micro Electro Mechanical Systems (MEMS 2014): IEEE; 2014. p. 1225–8.

[16] Menon L, Yang H, Cho S, Mikael S, Ma Z, Reuterskiöld-Hedlund C, et al Heterogeneously Integrated InGaAs and Si Membrane Four-Color Photodetector Arrays. IEEE Photonics Journal. 2016;8(2):1–7.

[17] Palmer TA, Alexay CC, Vogel S. Somewhere under the rainbow: The Visible to Far Infrared Imaging Lens. In: Andresen BF, Fulop GF, Norton PR, editors.;Vol SPIE-801223; Bellingham, WA: The International Society for Optical Engineering; 2011 p. 1–7.

[18] Wolff LB, Socolinsky DA, Eveland CK. Advances in low-power visible/thermal IR video image fusion hardware In: Peacock GR, Burleigh DD, Miles JJ, editors. Thermosense XXVII;Vol SPIE-5782 Bellingham, WA, USA: The International Society for Optical Engineering; 2005 p. 54–8.

[19] Angel H, Ste-Croix C, Kittel E. Review of fusion systems and contributing technologies for SIHS. Guelph, Ontario, Canada: Humansystems Inc., 2007 Contract No.: W7711-067989-05. https://www.researchgate.net/publication/235157386.

[20] Dwyer DJ, Smith MI, Dale JL, Heather JP. Real-time implementation of image alignment and fusion In: Dasarathy BV, editor. Multisensor, Multisource Information Fusion: Architectures, Algorithms, and Applications 2005;Vol SPIE-5813 Bellingham, WA, USA: The International Society for Optical Engineering; 2005 p. 85–93.

[21] Dwyer DJ, Hickman D, Riley T, Heather J, Smith M. Real time implementation of image alignment and fusion on a police helicopter In: Verly JG, Guell JJ, editors. Enhanced and Synthetic Vision 2006;Vol SPIE-6226 Bellingham, WA, USA: The International Society for Optical Engineering; 2006 p. 6226071–11.

[22] Riley T, Smith MI. Image fusion technology for security and surveillance applications In: Lewis C, Owen GP, editors. Optics and Photonics for Counterterrorism and Crime Fighting II;Vol SPIE-6402 Bellingham,WA, USA: The International Society for Optical Engineering; 2006 p. 6402041–12.

[23] Zou X, Bhanu B. Tracking humans using multi-modal fusion. 2nd Joint IEEE International Workshop on Object Tracking and Classification in and Beyond the Visible Spectrum (OTCBVS'05): IEEE; 2005. p. W01-30-1-8.

[24] O'Brien MA, Irvine JM. Information fusion for feature extraction and the development of geospatial information. 7th International Conference on Information Fusion: ISIF; 2004. p. 976–82.

[25] Toet A. Color image fusion for concealed weapon detection In: Carapezza EM, editor. Sensors, and command, control, communications, and intelligence (C3I) technologies for homeland defense and law enforcement II;Vol SPIE-5071 Bellingham, WA., USA: SPIE; 2003 p. 372–9.

[26] Xue Z, Blum RS. Concealed weapon detection using color image fusion. Sixth International Conference on Information Fusion (FUSION 2003); Cairns, Queensland, Australia: IEEE; 2003. p. 622–7.

[27] Xue Z, Blum RS, Li Y. Fusion of visual and IR images for concealed weapon detection. Fifth International Conference on Information Fusion;Vol 2; Annapolis, MD, USA: IEEE; 2002. p. 1198–205.

[28] Bhatnagar G, Wu QMJ. Human visual system based framework for concealed weapon detection. The 2011 Canadian Conference on Computer and Robot Vision (CRV); St. Johns, NL: IEEE; 2011. p. 250–6.

[29] Liu Z, Xue Z, Blum RS, Laganiëre R. Concealed weapon detection and visualization in a synthesized image. Pattern Analysis & Applications. 2006;8(4):375–89.

[30] Yajie W, Mowu L. Image fusion based concealed weapon detection. International Conference on Computational Intelligence and Software Engineering 2009 (CiSE 2009); Wuhan, China: IEEE; 2009. p. 1–4.

[31] Beyan C, Yigit A, Temizel A. Fusion of thermal- and visible-band video for abandoned object detection. Journal of Electronic Imaging. 2011;20(033001):1–12.

[32] Lepley JJ, Averill MT. Detection of buried mines and explosive objects using dual-band thermal imagery In: Harmon RS, Holloway JH, Broach JT, editors. Detection and Sensing of Mines, Explosive Objects, and Obscured Targets XVI;Vol SPIE-8017 Bellingham, WA, USA: The International Society for Optical Engineering; 2011 p. 80171V1–12.

[33] Kong SG, Heo J, Boughorbel F, Zheng Y, Abidi BR, Koschan A, et al Multiscale fusion of visible and thermal IR images for illumination-invariant face recognition. International Journal of Computer Vision. 2007;71(2):215–33.

[34] Liu Z, Liu C. Fusion of color, local spatial and global frequency information for face recognition. Pattern Recognition. 2010;43(8):2882–90.

[35] Estrera JP, Ostromek TE, Isbell W, Bacarella AV. Modern night vision goggles for advanced infantry applications In: Rash CE, Reese CE, editors. Helmet- and Head-Mounted Displays VIII: Technologies and Applications;Vol SPIE-5079 Bellingham, WA., USA: The International Society for Optical Engineering; 2003 p. 196–207.

[36] Estrera JP. Digital image fusion systems: color imaging and low-light targets In: Andresen BF, Fulop GF, Norton PR, editors. Infrared Technology and Applications XXXV;Vol SPIE-7298 Bellingham, WA, USA: The International Society for Optical Engineering; 2009 p. 72981E1–11.

[37] Frim J, Bossi L, Tack D. Human factors considerations of IR sensors for the Canadian Integrated Soldier System Project (ISSP) In: Andresen BF, Fulop GF, Norton PR, editors. Infrared Technology and Applications XXXV;Vol SPIE-7298 Bellingham, WA, USA: The International Society for Optical Engineering; 2009 p. 72981H1–12.

[38] Zitová B, Beneš M, Blažek J. Image fusion for art analysis Computer Vision and Image Analysis of Art II;Vol SPIE-7869 Bellingham, WA, USA: The International Society for Optical Engineering; 2011 p. 7869081–9.

[39] Bulanona DM, Burks TF, Alchanatis V. Image fusion of visible and thermal images for fruit detection. Biosystems Engineering. 2009;103(1):12–22.

[40] Ghassemian H. A retina based multi-resolution image-fusion. IEEE International Geoscience and Remote Sensing Symposium (IGRSS2001); Washington, USA: IEEE; 2001. p. 709–11.

[41] Jiang D, Zhuang D, Huan Y, Fu J. Survey of multispectral image fusion techniques in remote sensing applications In: Zheng Y, editor. Image Fusion and Its Applications. Rijeka, Croatia: InTech Open; 2011 p. 1–22. www.intechopen.com/download/pdf/15838.

[42] Jacobson NP, Gupta MR. Design goals and solutions for display of hyperspectral images. IEEE Transactions on Geoscience and Remote Sensing. 2005;43(11):2684–92.

[43] Jacobson NP, Gupta MR, Cole JB. Linear fusion of image sets for display. IEEE Transactions on Geoscience and Remote Sensing. 2007;45(10):3277–88.

[44] Daneshvar S, Ghassemian H. MRI and PET image fusion by combining IHS and retina-inspired models. Information Fusion. 2010;11(2):114–23.

[45] Yongqiang Z, Lei Z, Quan P. Spectropolarimetric imaging for pathological analysis of skin. Applied Optics. 2009;48(10):D236–46. 19340114 10.1364/ao.48.00d236

[46] Zaidi M, Montandon M-L, Alav A. The clinical role of fusion imaging using PET, CT, and MR imaging. PET Clinics. 2009;3(3):275–91. 10.1016/j.cpet.2009.03.002 27156662

[47] Rojas GM, Raff U, Quintana JC, Huete I, Hutchinson M. Image fusion in neuroradiology: three clinical examples including MRI of Parkinson disease. Computerized Medical Imaging and Graphics. 2006;31(1):17–27. 10.1016/j.compmedimag.2006.10.002 17150328

[48] Ukimura O. Image Fusion: InTech Open; 2011 http://www.intechopen.com/books/image-fusion.

[49] Li S, Kang X, Fang L, Hu J, Yin H. Pixel-level image fusion: A survey of the state of the art. Information Fusion. 2017;33:100–12.

[50] Bavirisetti DP, Dhuli R. Two-scale image fusion of visible and infrared images using saliency detection. Infrared Physics & Technology. 2016;76:52–64.

[51] Bavirisetti DP, Dhuli R. Fusion of infrared and visible sensor images based on anisotropic diffusion and Karhunen-Loeve transform. IEEE Sensors Journal. 2016;16(1):203–9.

[52] Bhatnagar G, Liu Z. A novel image fusion framework for night-vision navigation and surveillance. Signal, Image and Video Processing. 2015;9(1):165–75.

[53] Gan W, Wu X, Wu W, Yang X, Ren C, He X, et al Infrared and visible image fusion with the use of multi-scale edge-preserving decomposition and guided image filter. Infrared Physics & Technology. 2015;72:37–51.

[54] Kong W, Wang B, Lei Y. Technique for infrared and visible image fusion based on non-subsampled shearlet transform and spiking cortical model. Infrared Physics & Technology. 2015;71:87–98.

[55] Li H, Qiu H, Yu Z, Zhang Y. Infrared and visible image fusion scheme based on NSCT and low-level visual features. Infrared Physics & Technology. 2016;76:174–84.

[56] Ma J, Chen C, Li C, Huang J. Infrared and visible image fusion via gradient transfer and total variation minimization. Information Fusion. 2016;31:100–9.

[57] Meng F, Guo B, Song M, Zhang X. Image fusion with saliency map and interest points. Neurocomputing. 2016;177:1–8.

[58] Zhang X, Yu L, Huang G. Infrared and visible image fusion based on shearlet transform and image enhancement. Seventh International Conference on Digital Image Processing (ICDIP 2015);Vol SPIE-9631. Bellingham, WA: SPIE; 2015. p. 96310I-6.

[59] Yan X, Qin H, Li J, Zhou H, Zong Jg. Infrared and visible image fusion with spectral graph wavelet transform. Journal of the Optical Society of America A. 2015;32(9):1643–52. 10.1364/JOSAA.32.001643 26367432

[60] Zhao J, Gao X, Chen Y, Feng H, Wang D. Multi-window visual saliency extraction for fusion of visible and infrared images. Infrared Physics & Technology. 2016;76:295–302.

[61] Zhou Z, Wang B, Li S, Dong M. Perceptual fusion of infrared and visible images through a hybrid multi-scale decomposition with Gaussian and bilateral filters. Information Fusion. 2016;30(1):15–26.

[62] Zhou Z, Dong M, Xie X, Gao Z. Fusion of infrared and visible images for night-vision context enhancement. Applied Optics. 2016;55(23):6480–90. 10.1364/AO.55.006480 27534499

[63] Toet A. Iterative guided image fusion. PeerJ Computer Science. 2016;2(e80):1–26.

[64] Schaul L, Fredembach C, Süsstrunk S. Color image dehazing using the near-infrared. Procedings of the IEEE International Conference on Image Processing (ICIP2009); Cairo, Egypt: IEEE Press; 2009. p. 1629–32.

[65] Fredembach C, Süsstrunk S. Colouring the near-infrared. IS&T/SID 16th Color Imaging Conference; Springfield, VA: The Society for Imaging Science and Technology; 2008. p. 176–82.

[66] Shibata T, Tanaka M, Okutomi M. Versatile visible and near-infrared image fusion based on high visibility area selection. Journal of Electronic Imaging. 2016;25(1):013016-.

[67] Yu X, Ren J, Chen Q, Sui X. A false color image fusion method based on multi-resolution color transfer in normalization YCBCR space. Optik—International Journal for Light and Electron Optics. 2014;125(20):6010–6.

[68] Toet A. Natural colour mapping for multiband nightvision imagery. Information Fusion. 2003;4(3):155–66.

[69] Ulhaq A, Yin X, He J, Zhang Y. FACE: Fully automated context enhancement for night-time video sequences. Journal of Visual Communication and Image Representation. 2016;40, Part B:682–93.

[70] Jiang M, Jin W, Zhou L, Liu G. Multiple reference images based on lookup-table color image fusion algorithm. International Symposium on Computers & Informatics (ISCI 2015): Atlantis Press; 2015. p. 1031–8.

[71] Ali EA, Qadir H, Kozaitis SP. Color night vision system for ground vehicle navigation. In: Andresen BF, Fulop GF, Hanson CM, Norton PR, editors.;Vol SPIE-9070-0I; Bellingham, MA,USA: SPIE; 2014 p. 1–5.

[72] Zheng Y, Reese K, Blasch E, McManamon P. Qualitative evaluations and comparisons of six night-vision colorization methods. In: Kadar i, Kadar I, editors.;Vol SPIE-8754; Bellingham, MA, USA: The International Society for Optical Engineering; 2013 p. 874511-.

[73] Qu Z, Xiao G, Xu N, Diao Z, Jia-Zhou H. A novel night vision image color fusion method based on scene recognition. 19th International Conference on Information Fusion (FUSION); 5–8 July 2016; Heidelberg, Germany: IEEE; 2016. p. 1236–43.

[74] Toet A. TNO Image fusion dataset. 2014. 10.6084/m9.figshare.1008029.

[75] Toet A. Kayak image fusion sequence. 2014. 10.6084/m9.figshare.1007650

[76] Liu Z, Blasch E, Xue Z, Zhao J, Laganiere R, Wu W. Objective assessment of multiresolution image fusion algorithms for context enhancement in night vision: A comparative study. IEEE Transactions on Pattern Analysis and Machine Intelligence. 2012;34(1):94–109. 10.1109/TPAMI.2011.109 21576753

[77] Choi Y, Kim N, Park K, Hwang S, Yoon J, Kweon I. All-day visual place recognition: Benchmark dataset and baseline. IEEE International Conference on Computer Vision and Pattern Recognition Workshops (CVPRWVPRICE); Boston, MA, USA. 8–10 June 2015. p. 8–10.

[78] Toet A, Hogervorst MA. TRICLOBS portable triband lowlight color observation system In: Dasarathy BV, editor. Multisensor, Multisource Information Fusion: Architectures, Algorithms, and Applications 2009;Vol SPIE-7345 Bellingham, WA, USA: The International Society for Optical Engineering; 2009 p. 7345031–11.

[79] Hogervorst MA, Toet A. Fast natural color mapping for night-time imagery. Information Fusion. 2010;11(2):69–77.

[80] Toet A, Hogervorst MA, van Son R, Dijk J. Augmenting full color fused multiband night vision imagery with synthetic imagery for enhanced situational awareness. International Journal of Image and Data Fusion. 2011;2(4):287–308.

[81] Toet A, Hogervorst MA, Dijk J, van Son R. INVIS: integrated night vision surveillance and observation system In: Güell JJ, Bernier KL, editors. Enhanced and Synthetic Vision 2010;Vol SPIE-7689 Bellingham, WA, USA: The International Society for Optical Engineering; 2010 p. 7689061–16.

[82] Toet A. INVIS: Integrated night vision surveillance and observation system 2015. 10.6084/m9.figshare.1495334.v1.

[83] Toet A. Presentation of the INVIS full color night vision system 2015. 10.6084/m9.figshare.1495335.v1.

[84] Hogervorst MA, Toet A. Evaluation of a color fused dual-band NVG In: Dasarathy BV, editor. Multisensor, Multisource Information Fusion: Architectures, Algorithms, and Applications 2009;Vol SPIE-734502 Bellingham, WA: The International Society for Optical Engineering; 2009 p. 1–7.

[85] Hogervorst MA, Toet A. Presenting nighttime imagery in daytime colours. 11th International Conference on Information Fusion; Cologne, Germany: IEEE; 2008. p. 706–13.

[86] Toet A, Hogervorst MA. Progress in color night vision. Optical Engineering. 2012;51(1):010901-1-19.

[87] Hogervorst MA, Jansen C, Toet A, Bijl P, Bakker P, Hiddema AC, et al Colour-the-INSight: combining a direct view rifle sight with fused intensified and thermal imagery In: Braun JJ, editor. Information Systems and Networks: Processing, Fusion, and Knowledge Generation;Vol SPIE-8407-24; Bellingham, WA: The International Society for Optical Engineering; 2012 p. 1–10.

[88] Toet A, Hogervorst MA. Real-time full color multiband night vision In: Gallegos-Funes F, editor. Vision Sensors and Edge Detection. Rijeka, Croatia: INTECHopen; 2010 p. 105–42. www.intechopen.com/download/pdf/11883.

[89] Heckbert P. Color image quantization for frame buffer display. Computer Graphics. 1982;16(3):297–307.

[90] Ruderman DL, Cronin TW, Chiao C-C. Statistics of cone responses to natural images: implications for visual coding. Journal of the Optical Society of America A. 1998;15(8):2036–45.

[91] Burt PJ, Adelson EH. The Laplacian pyramid as a compact image code. IEEE Transactions on Communications. 1983;31(4):532–40.

[92] Toet A. Hierarchical image fusion. Machine Vision and Applications. 1990;3(1):1–11.

[93] Pinkus AR, Dommett DW, Task HL. A comparison of Landolt C and triangle resolution targets using the synthetic observer approach to sensor resolution assessment Signal Processing, Sensor Fusion, and Target Recognition XXI;Vol SPIE-83921A The International Society for Optical Engineering; 2012 p. 1–9.

[94] Pinkus AR, Dommett DW, Task HL. A comparison of sensor resolution assessment by human vision versus custom software for Landolt C and triangle resolution targets In: Kadar I, editor. Signal Processing, Sensor Fusion, and Target Recognition XXII;Vol SPIE-8745 Bellingham, WA, USA: The International Society for Optical Engineering; 2013 p. 87450Z1–12.

